# Application of Clove Oil and Sonication Process on the Influence of the Functional Properties of Mung Bean Flour-Based Edible Film

**DOI:** 10.3390/membranes12050535

**Published:** 2022-05-20

**Authors:** Ittiporn Keawpeng, Somwang Lekjing, Balaji Paulraj, Karthikeyan Venkatachalam

**Affiliations:** 1 Faculty of Agricultural Technology, Songkhla Rajabhat University, Muang, Songkhla 90000, Thailand; ittiporn.ke@skru.ac.th; 2 Faculty of Innovative Agriculture and Fishery Establishment Project, Prince of Songkla University, Surat Thani Campus, Makham Tia, Muang, Surat Thani 84000, Thailand; somwang.s@psu.ac.th; 3 PG and Research Centre in Biotechnology, MGR College, Hosur 635130, Tamil Nadu, India; balaji_paulraj@yahoo.com

**Keywords:** mung bean flour, edible films, clove oil, sonication, antioxidant and antimicrobial properties

## Abstract

The present study was aimed to investigate the effects of sonication and clove oil incorporation on the improvement of physical, antioxidant, and antimicrobial properties and lipid oxidation inhibiting abilities of mung bean flour (MF)-based films. There were three groups of films tested (1) MF: mung bean flour alone, (2) MFC: MF incorporated with 2% clove oil (C), and (3) MFCU: MFC prepared with sonication (25 kHz, 100% amplitude, 10 min). Film thickness and bulk density showed slight differences, and moisture content, solubility, and water vapor permeability significantly differed between the formulations. Tensile strength, elongation at break, and Young’s modulus were highest for the MFCU films, followed by MFC and MF in rank order. Furthermore, the Fourier-transform infrared spectroscopy results also demonstrated that the clove oil and sonication treatment had improved the interconnections of the biopolymers, thus increasing the physical strength of the film. Phytochemicals in terms of total phenolics and total flavonoids were elevated in the MFCU films and contributed to stronger radical scavenging abilities (*p* < 0.05). MFC and MFCU films showed a strong antibacterial control of the Gram-positive *Staphylococcus aureus* (*S. aureus*) and also of the Gram-negative *Campylobacter jejuni* (*C. jejuni*). Overall, the lipid oxidation indicators Thiobarbituric acid reactive substances (TBARS, peroxide value, p-anisidine value, and totox value) showed significantly high inhibition, attributed to radical scavenging activities in the MFCU and MFC samples. The mung bean flour films incorporated with clove oil and prepared with sonication have good potential as packaging materials for food due to strong physical, antimicrobial, and antioxidant properties, as well as lipid oxidation inhibiting abilities.

## 1. Introduction

Legumes are the third largest family in the plant kingdom and consist of 18,000 species. Among them, only 200 species are considered safe for use in food or feed [1]. Mung bean (*Vigna radiata* L.) is one of the staple legumes produced widely in Asian countries, especially in Thailand. It is also known by many names, including green gram, moong bean, and thuvar kiaw [2]. Mung bean is well known as a high nutritious legume that is composed mainly of protein (25–28%), carbohydrates (55–65%), and lipids (1–3%), and the other constituents include vitamins, minerals, and polyphenols (7–24%) [3]. The composition of mung bean varies depending on how its processing: the raw bean has more nutritional content than cooked or sprouted ones. Bioactive compounds from the mung bean known as secondary metabolites include flavonoids, phenolic acids, organic acids, sterols, triterpenes, and aldehydes, which possess various potential pharmacological effects [4]. Studies have reported that flavonoids and organic acids are abundant in the mung bean, and so far, 15 flavonoids and 21 organic acids have been identified and characterized [5,6]. Furthermore, these compounds are believed to be the major components in the mung bean that modulate human immune responses and to have other pharmacological activities (antioxidant, antimicrobial, antidiabetic, antilipidemic, anti-carcinogenic, antitumor, and detoxifying agent) [7,8,9]. A variety of food products are produced using the mung bean for short-term consumption and long-term consumption. Aside from food products, the high contents in mung bean of protein, polysaccharides, and phytochemicals could support developing other natural and eco-friendly products.

Food packaging plays a critical role in the food industry as it preserves food materials shielding the consumers from various hazards [10]. Synthetic packaging materials play an extraordinary role in the convenience of our daily lives; however, since they are not biodegradable, they negatively impact ecology and the environment, including pollution of the marine environment that can take several decades to rectify [11]. Recently, the development of eco-friendly biodegradable and edible polymeric packages from natural food raw materials has been widely explored to optimize their characteristics and applications [12]. A biodegradable polymer is formed by combining biodegradable and natural functional groups in its structure, it can easily be degraded by fungi, algae, and bacteria, and can decompose into water, biomass, CO_2_, and methane [13,14]. In recent times, active biodegradable packaging films have been thriving as they can incorporate various bioactive ingredients or essential oils to improve structural integrity and barrier, as well as antioxidant and antimicrobial properties. The use of biodegradable polymers in manufacturing consumes less energy than plastics, and also makes them more environmentally friendly because of their renewable resources. Furthermore, biodegradable polymers from edible protein and starch bases are a weak barrier to moisture, and therefore, incorporation of lipid-based additives are recommended for hydrophobicity and flexibility [14]. Clove bud essential oil (CO) is an important bioactive ingredient widely applied in edible films [15]. Eugenol is the predominant active ingredient in clove oil and is responsible for various pharmacological effects. To obtain films, homogenization is crucial to form an emulsion, and it significantly improves the microstructural stability of the eventual film. Small droplets and high emulsion stability are the key factors enabling a homogenous film matrix and good crosslinking of the polymer networks [16]. Fattahi et al. [17] found that sonication as a high-energy homogenization technique, strengthens the mechanical and antimicrobial properties of films incorporating essential oils. Yildiz et al. [18] found that pea flour-based edible film, when treated with sonication, had significantly increased crosslinking of the biopolymers and improved physical properties.

A protein-based film with added polysaccharides can provide good mechanical strength and excellent gas and moisture barrier properties by its tightly packed network structure [19]. Naturally, mung bean is an excellent source of proteins and carbohydrates, and it is also an excellent source of hydrocolloids (carrageenan, locust bean gum, and xanthan gum), and it is also capable of forming edible films [20]. Moghadam et al. [3] reported that active film based on protein-rich mung bean flour incorporating pomegranate peel exhibited good antioxidant and antimicrobial properties. Janikova et al. [21] observed that addition of natural extracts, particularly from *Clitoria ternatea*, *Brassica oleracea*, and *Ipomea batatas*, in the protein-polysaccharide based edible film emulsion and treatment with fresh cut apples was highly effective in controlling the surface browning and significantly extended their shelf life. To the best of our knowledge, no prior study is available on the fabrication of mung bean flour-based edible film emulsion with clove essential oil and homogenization using sonication to develop biodegradable edible films with strong physical, biochemical, and functional properties. The present study examined the physicochemical properties, antioxidant and antimicrobial properties, and lipid oxidation inhibiting abilities of mung bean flour film incorporating clove essential oil and prepared with sonication.

## 2. Materials and Methods

### 2.1. Materials

Mung beans were purchased from a local market and processed into flour by using the method of Venkatachalam et al. [2]. For flour preparation, mung beans were sorted, cleaned (destoned), and then ground separately using an electric grain mill, then sieved manually using a 44-mesh sieve before being used. Mung bean flour should then be stored in an airtight container at ambient temperature until use (all flour should be used within a week after it has been prepared). The proximate compositions of the mung bean flour were tested in accordance with the method of Association of Official Analytical Chemists (AOAC) [22], and the obtained results are as follows: 61% carbohydrate, 22% protein, 1.5% fat, 9.6% moisture, 2.78% fiber, and 3.12% ashes. CO (>95%) and glycerol (99%) were purchased from a local supplier in Thailand. All other chemicals and reagents (Tween 80 (10%), gallic acid (97.5%), Folin–Ciocalteu’s phenol reagent (55%), sodium carbonate (99.5%), sodium nitrite (97%), aluminum chloride (99%), sodium hydroxide (98%), 2,2-Di(4-tert-octylphenyl)-1-picrylhydrazyl, free radical (85%), catechin (97%), 2,2′-Azino-bis(3-ethylbenzothiazoline-6-sulfonic acid) diammonium salt (95%), ferrous ammonium sulfate (99%), ethylenediamine tetra acetic acid (99.4%), dimethyl sulfate (95%), ascorbic acid (99%), trichloro acetic acid (99%), Nash reagent (60%), 2-thiobarbituric acid (98%) and malondialdehyde (96%)) were purchased from Sigma Aldrich, Singapore. Media for microbial analysis were procured from HiMedia Laboratories, Mumbai, India.

### 2.2. Methods

#### 2.2.1. Film Formation

**Figure 1:**
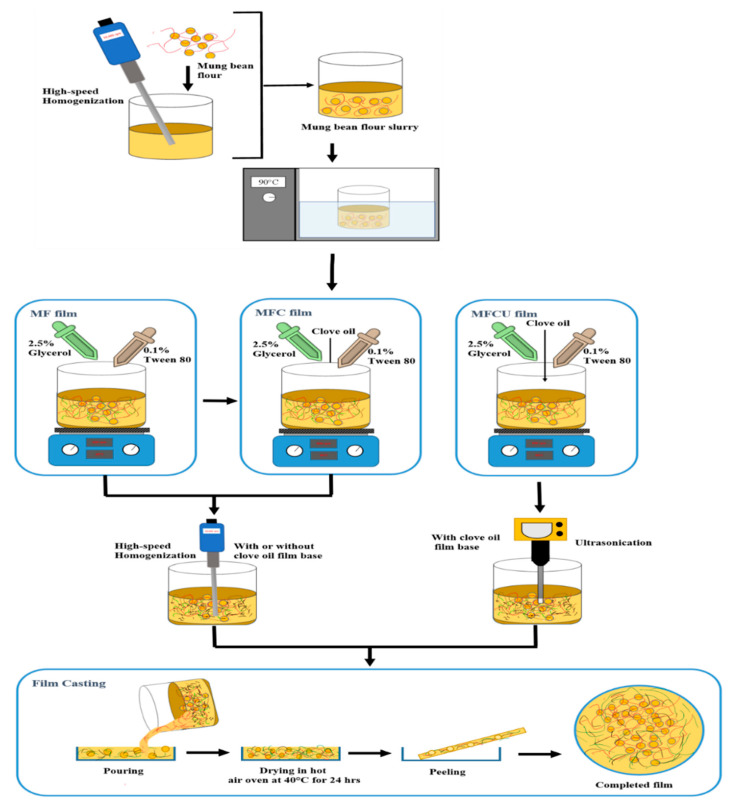
Schematic representation of mung bean flour film formation and its variations. Note MF represents mung bean flour films; MFC represents mung bean flour film incorporated with clove oil; MFCU represents the mung bean flour film incorporated with clove oil and processed with sonication.

Mung bean films were prepared using a casting method. A 10% mix of mung bean flour with distilled water (*w*/*v*) was prepared using a high-speed homogenizer at 10,000 rpm for 10 min. Then, the slurry pH was adjusted to 10 ± 0.02 by using NaOH (0.1 M). Next, the slurry was gelatinized for 30 min in a water bath regulated to 90 °C. Later, the slurry was removed from the water bath and allowed to cool down to 40 °C at the ambient temperature while stirring at 500 rpm using a magnetic stirrer. Then, the slurry was incorporated with 2.5% glycerol (*w*/*v*) and with 0.1% Tween 80 (*w*/*v*), with or without 0.75% of clove bud essential oil. To see effects of homogenization, the slurry was homogenized using two alternative methods: in the first method, the slurry was homogenized using the high-speed homogenizer (10,000 rpm for 10 min); and in the second method, the slurry was homogenized using ultrasonication (Hielscher UP200Ht, Teltow, Germany), in which an ultrasound probe was submerged into the slurry mix and operated at 200 W power for 10 min at 95% amplitude. Then, an ultrasonication bath (25 kHz for 20 min) was used to remove any air bubbles in the slurry. For film-forming, a 15 mL sample of the processed slurry was evenly poured into a Petri dish and dried at 40 °C in a hot air oven (Binder, Tuttlingen, Germany) for 24 h. Subsequently, the plates were left in a desiccator (50% relative humidity) for 24 h at ambient temperature before proceeding to the various analyses, and the films were maintained in this same condition until all the quality analysis was done. The scheme of film formation process is shown in Figure 1.

#### 2.2.2. Quality Analysis

##### Physical Properties

The film thickness of the mung bean films was measured using a handheld digital micrometer (Mitutoyo 293-340-30 External Micrometer, Kawasaki, Japan). For each treatment, ten films were used, and each film was tested for thickness at six random sites. The results are expressed in millimeters. The bulk density of the mung bean films was measured by following the Venkatachalam et al. [2] method with slight modifications. Films were measured for thickness at random points, and then they were dried in a hot air oven for 24 h at 40 °C or until constant weight. After that, the bulk density was measured based on the ratio of the final dry weight to the volume (thickness of film x area). The results are expressed as g/cm^3^. Moisture content in the films was measured using a digital infrared moisture analyzer. The results are expressed as percentages. The solubility of the films was tested based on the method of Aydogdu et al. [23]. Prior to the test of the solubility, the films were cut into squares (2.5 cm × 2.5 cm) and dried at 40 °C using a hot air oven for 24 h or until the films reached a constant weight (W_o_). Then, the films were submerged in a beaker containing 50 mL distilled water for 24 h at ambient temperature. Afterward, the solution was filtered, and the retained solids were dried in an oven at 105 °C for 24 h, and the final weight (W_f_) was recorded. Then, the Equation (1) was used to calculate the solubility.

Solubility (%) = [(W_o_ − W_f_)/Wo] × 100 (1)

The water vapor permeability of the films was measured based on the gravimetric modified cup method. The cylindrical test cups (40 mm internal diameter) were filled with 35 mL distilled water to ensure 100% relative humidity in the cups. Film thickness was randomly measured at six different spots on the film using a handheld digital micrometer (Mitutoyo 293-340-30 External Micrometer, Kawasaki, Japan). Then, the film was set on the cup, the cap was used to provide a taut surface and seal the sides, and the set-up was left to allow the water vapor to permeate through the film. The initial weight was recorded, and then the cups were placed in a pre-equilibrated desiccator (10–25% relative humidity) using silica gel. Then, the changes in the cup weight, relative humidity, and temperature in the desiccator were monitored and recorded every 2 h for 24 h. The Formula (2) was used to calculate the water vapor permeability (WVP) of the film:

WVP (Kg Pa^−1^ s^−1^ m^−1^) = (G × x)/[ t × A × S × (R_1_ − R_2_)] (2)

where G represents water vapor flow (kg), x is film thickness (m), t is time (s), A is the area (m^2^), S is saturated WVP (Pa) at the measured temperature, R_1_ is the relative humidity inside the cup, and R_2_ is the relative humidity inside the desiccator.

##### Mechanical Properties

The mechanical properties tensile strength, elongation at break (EAB), and Young’s modulus of the films were measured using a texture analyzer. Prior to analysis, the films were cut into small-sized (2 cm × 6 cm) samples, and then the tensile strength was determined with 0.1 N preload at a fixed test speed of 0.30 mm/s. The measurement run ended when the film split into two pieces. The tensile strength was calculated as the maximum load’s ratio to the film’s cross-sectional area, and the results are expressed in MPa. EAB was calculated as the ratio of the length of film at the break to the film’s initial length (6 cm), and the results are expressed as percentages. Young’s modulus was determined from the stress-stress curve and is expressed in MPa.

##### Fourier Transform Infrared (FTIR) Spectroscopy

Films were subjected to FTIR spectroscopy (Thermo Electron Corp., Madison, WI, USA) using the method of Lekjing and Venkatachalam (2020). Films were mixed with potassium bromide in a 1:100 ratio, and the mixture was ground into powder and then pressed into a pellet. Then, the films were scanned to assess possible interactions between clove oil, glycerol, and mung bean flour, and the influences of sonication, by scanning 16 times within the wavelength range of 500–3500 cm^−1^ with 2 cm^−1^ resolution for each recorded spectrum.

##### Antimicrobial Activity

The film’s antimicrobial activity [24] was tested against the two microorganisms, namely Gram-positive *S. aureus* and Gram-negative *C. jejuni*. A film disc (1.5 cm diameter) was submerged in a glass tube that contained 10 mL of tryptic soy broth (TSB) and brain heart infusion broth (BHIB) and 1 mL of Gram (+) (in TSB medium) or Gram (−) (in BHIB medium) bacteria was added and mixed. Then, the tubes with *S. aureus* were transferred to the shaker and shaken at 200 rpm at room temperature for 24 h, whereas the tubes that contained *C. jejuni* were incubated for 48 h at 42 °C in microaerophilic conditions. After incubation, 1 mL microbial aliquots from each tube were collected and serially diluted, for which buffered peptone water was used with *S. aureus*, and BHIB medium was used with *C. jejuni*. Then, *S. aureus* (initial CFU/mL was at 7.14 ± 0.13) was plated on Mueller–Hinton agar plates and incubated for 24 h at 37 °C, whereas *C. jejuni* (initial CFU/mL was at 7.93 ± 0.13) was plated on *Campylobacter* agar plates and incubated for 48 h at 42 °C. After incubation, the colonies were counted, and the results were expressed in log CFU/mL.

##### Phytochemicals and Antioxidant Activities

Films were extracted to determine their phytochemical and antioxidant activities. First, a 1 g film sample was homogenized with 15 mL of 80% ethanol, and the homogenate was centrifuged (HERMLE Labortechnik GmbH, Z36 HK, Wehingen, Germany) at 6000× *g* for 15 min. After centrifugation, the clear supernatant was collected and used for the following analyses.

For total phenolic content [25], 100 µL of supernatant was added into the test tube, followed by the addition of 8.4 mL of distilled water and 500 µL of Folin–Ciocalteu reagent. Then, 1 mL of sodium carbonate (20%) was added and mixed well. Then, the reaction mixture was incubated at room temperature for 30 min and measured using a spectrophotometer at 720 nm. The absorbance of the sample was compared with the calibration curve for gallic acid [10–100 µg/ mL; R^2^ = 0.9970; *p* < 0.0001]. The results are expressed in µg gallic acid equivalents per sample volume (GAE)/mL. For total flavonoid content [22], 250 µL of supernatant was added into the test tube, and then 2.72 mL of 30% ethanol and 120 µL of 0.5 mol/L sodium nitrite were added and mixed well and allowed to stand for 5 min. Then, 120 µL of 0.3 mol/L aluminum chloride was added to the mixture, followed by adding 800 µL of 1 mol/L sodium hydroxide and mixing well. After that, the reaction mixture was measured at 510 nm using a spectrophotometer. The absorbance of the sample was compared with the calibration curve for catechin [10–100 µg/ mL; R2 = 0.9960; *p* < 0.0001]. The results are expressed in µg catechin equivalents per sample volume, (CE)/mL.

For DPPH (2,2-diphenyl-1-picrylhydrazyl) radical scavenging assay [26], 100 µL of supernatant was added to the test tube containing 3.9 mL of 60 µmol/L DPPH that prepared with 95% ethanol and mixed well. Subsequently, the reaction mixture was incubated for 30 min in the dark at ambient temperature. Then, the reaction mixture was measured at 515 nm using a spectrophotometer. The results are expressed as percentages of DPPH radical scavenging ability. For ABTS (2,2′-azino-di-3-ethylbenzthiazoline sulfonic acid) radical cation scavenging assay, a 100 µL of supernatant was added to a test tube and mixed with 100 µL ABTS reagent (as described in Lee et al. [27]) in a 96-well microplate, and then it was incubated for 6 min at room temperature. After incubation, the sample was measured at 734 nm using a microplate reader. The results are expressed as percentages of ABTS radical scavenging ability. For FRAP (ferric reducing antioxidant potential assay), 100 µL of supernatant was mixed with 3 mL of FRAP reagent (as described in Alberti et al. [25]). The reaction mixture was incubated for 20 min to develop a blue-colored complex, and then it was measured at 593 nm using a spectrophotometer. The results are expressed as percentages of ferric reducing antioxidant potential. For hydroxyl radical scavenging assay [28], 1 mL of WACV was added in a test tube containing 1 mL of ferrous ammonium sulfate (0.13%), ethylenediamine tetra acetic acid (EDTA) (0.26%) solution, 0.5 mL of 0.018% EDTA, 1 mL of 0.85% dimethyl sulfoxide and 0.22% ascorbic acid. After that, the reaction mixture was mixed well and incubated in a water bath at 90°C for 10 min. Then, the reaction was terminated by adding 1 mL of ice-cold trichloroacetic acid (TCA), followed by 3 mL of Nash reagent wax, and then was mixed in the reaction mixture and kept at room temperature for 15 min to develop a yellow color. Then, the reaction mixture was measured at 412 nm using a spectrophotometer. The results are expressed as percentages of hydroxyl radical scavenging ability.

##### Lipid Oxidation Inhibition via Radical Scavenging

To test for inhibition of lipid oxidation (caused by radical scavenging ability of the film), 25 mL of olive oil was placed into the glass tube, and 0.5 g of the film was added into the tube and mixed well. Then the tube was kept in the dark at ambient temperature for 15 days to observe inhibition of lipid oxidation. At 3-day intervals, oil samples were collected and measured for thiobarbituric acid reactive substance (TBARS), peroxide value, p-anisidine value, and totox value. TBARS in the oil sample was measured in accordance with the method of Venkatachalam and Lekjing [15]. The absorbance of the sample was compared with the standard curve for malondialdehyde (MDA). The results are expressed as mg MDA/g oil.

#### 2.2.3. Statistical Analysis

In this study, all the experiments were done in triplicates and the data are presented as mean ± standard deviation. The comparison between the means were tested and performed at a probability level of at *p* < 0.05 using a one-way ANOVA (analysis of variance) followed by Tukey’s Multiple Comparison Test. All the statistical analysis in this study was carried out by using the Statistical Package for the Social Sciences (SPSS) software version 12 for Windows.

## 3. Results and Discussion

### 3.1. Physical Properties

Table 1 shows the physical properties of thickness, bulk density, moisture content, solubility, and water vapor permeability for films from mung flour incorporated with clove oil and processed with ultrasonication. Both clove oil and ultrasonication treatment significantly influenced the mung bean flour film’s physical properties. Film thickness was between 0.191 and 0.198 mm, and differences in film thickness between the control and the treatments were not significant. However, slight changes were noted in the mung bean flour film with clove oil prepared sonication. The thickness of the tested films in this study is in accordance with typical packaging films used in the food industry. The mung bean flour film’s bulk density ranged within 0.86 g/cm^3^–0.97 g/cm^3^. The inclusion of clove oil and sonication treatment slightly increased the density of the film, but the differences were not statistically significant. Several studies have reported that the inclusion of oil in a flour-based film did not influence the density [29]. The film’s moisture content ranged from 7.45 to 12.46%, with the lowest values observed for the mung bean flour film incorporated with clove oil and processed with sonication (MFCU)films and the highest for MF. The control film shows that homogenization during film making might physically induce interactions with the water that retains it so that more moisture was observed in the film. The mung bean flour film incorporated with clove oil (MFC) and MFCU films were hydrophilic but possibly with less interaction with water, as suggested by the moisture results observed in this study. Furthermore, the sonication produced from the collapse of cavitation bubbles acoustic shocks that could help disintegrate molecules or micellar structures and decrease the water-holding capacity of the film [30]. The film’s moisture content could also be affected by the macromolecule (protein and starch) contents. The sonication could reduce the starch molecule sizes, consequently lowering the film’s water holding capacity [31]. Low water solubility is a desired property of the film, as it can then provide a longer shelf life. The present study showed that mung bean flour films, particularly MFC and MFCU, showed reduced water solubility. The MF films’ water solubility ranged between 28 and 31%. MFC and MFCU films exhibited a lower water solubility than the mung bean flour film (MF), and it could be due to the action of clove oil, which strongly incorporated within the film and increased the hydrophobicity of the film. Numerous studies have reported that films produced from legumes have high aqueous solubility (>40%) [32,33], and the present study is in contrast with those results. This might be due to the influences of processing conditions and raw material variations, which might play a significant role in the solubility differences. The water vapor permeability (WVP) of the mung bean films that were incorporated with clove oil and then processed with sonication were significantly different. Generally, a mixing procedure used in film preparation should form small-sized particles and thus reduce the films’ WVP, with a tight fine-scale structure obstructing vapor passage through the film. According to the Table 1, MFCU showed lower WVP than MFC. Plus, consider that the oil itself causes the film to be more hydrophobic, which results in a higher resistance to vapor water transfer. It could be due to the ultrasound treatment causing polymer scission and breakage of crosslinks in the starch component of the film [34,35].

### 3.2. Mechanical Properties

The mechanical properties of tensile strength, elongation at break, and Young’s modulus are crucial characteristics of an edible food packaging film. The MF film’s tensile strength was significantly affected by adding clove oil and sonication (Table 2). MF film had a lower tensile strength, which was improved with the incorporation of the essential oil and the sonication treatment. Significant differences were also noted between MFC and MFCU. The film’s tensile strength could be weakened if polar groups’ interactions increased through hydrogen bonding or due to weak intermolecular interactions. The present study indicates that adding clove oil increased the film’s hydrophobic nature, thus reducing the hydrophilic intermolecular interactions and promoting the film’s tensile strength. The increased tensile strength of MFCU film caused by sonication could be attributed to the increased hydration level of the starch, which then contributed to the strength of the films [35]. Elongation at break is one of the critical parameters associated with the film’s flexibility [36]. The present study showed that MF film prepared with clove oil and sonication showed an improved elongation tolerance to the film (Table 2). MF flour contained dominantly starch, followed by protein and other ingredients. Typically, the starch itself is less brittle and supports large elongation, and the addition of clove oil and sonication significantly improved the elongation properties of the film. Studies have found that phenolic compounds in the clove oil could be binding with the film’s polymer matrix. The sonication could expose the polymer matrix to the phenolics in clove oil and thus enable interactions observed as increased flexibility. Young’s modulus is a rigidity indicator of the film; increasing the modulus represents increased rigidity. The present study showed that adding clove oil decreased Young’s modulus of MF film (Table 2). Furthermore, sonication also improved the flexibility of the film and further lowered the modulus. The sonication could improve the biopolymers’ dispersibility and decrease particle size in the film components, thereby improving the film’s bendability and stretchability. The mechanical properties of MFC and MFCU are similar to commercial nylon packages and could be suitable coatings for the packaging of food products.

### 3.3. FTIR Absorbance Spectrum

**Figure 2:**
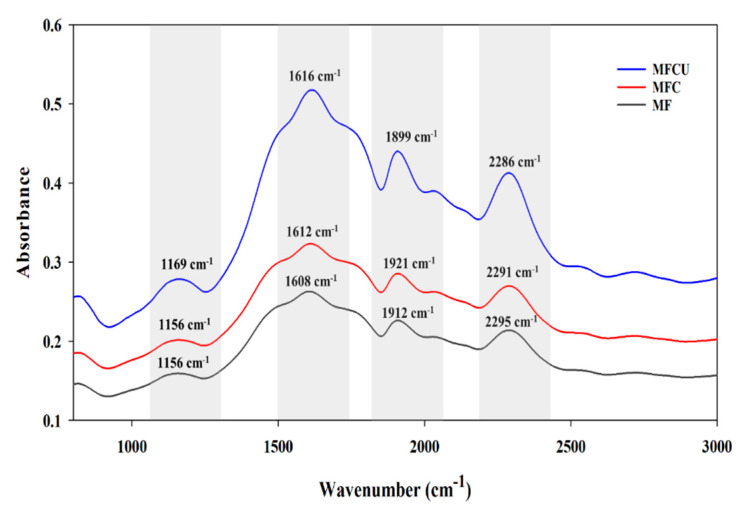
FTIR spectra of mung bean flour-based films processed under different conditions.

The FTIR absorbance spectra of the mung bean flour films are shown in Figure 2. The results demonstrate that the addition of clove oil and sonication increased the absorbance peak intensity compared to MF films. The peak intensities were highest for MFCU, followed by MFC and MF. The MF-based film showed characteristic peaks between 1300 and 2500 cm^−1^. The peaks around 2500 cm^−1^ with small variations in the peak height could be attributed to hydrogen bonds and hydroxyl groups. The peaks were slightly higher for MFC and MFCU, possibly due to the added clove oil that contributes hydroxyl groups in the phenolics, and the sonication might degrade the starch slightly, releasing more hydroxyl groups contributing to the peaks around 2300–2400 cm^−1^. The bands between 2300 and 2600 cm^−1^ indicate the presence of fat in the film. The clove oil contributed to those peaks in MFCU and MFC, while it was missing in the MF film with no clove oil. Stoleru et al. [37] reported that some peaks for the oil-containing film could be attributed to the stretching of carbonyl groups in clove oil. Strong peak bands were observed between 1400 and 2000 cm^−1^, representing proteins in the film, and some peaks could be attributed to stretching of amide groups (C = O and C-N) in protein and angular deformations N-H groups [24,38]. Furthermore, the peak observed between 1300 and 1400 cm^−1^ is due to asymmetric and symmetric deformations of -CH_3_ [39]. The starch content was responsible for the bands located between 1000 and 1300 cm^−1^, and similar peaks were found for all the films; however, they were slightly higher for MFCU and MFC. Warren et al. [40] reported that the bands between 900 and 1300 cm^−1^ were due to stretching and bending of C-O-H, and also due to the starch glycosidic linkages. Hoque et al. [41] reported that the peaks between 1000–1200 cm^−1^ were due to interactions in the film with the added plasticizer.

### 3.4. Phytochemical and Antioxidant Activities 

The phytochemical contents of TPC and TFC in the films are shown in Table 3. Overall, the films contained a richer level of TPC and TFC, and MFC and MFCU films had slightly higher levels of phytochemicals than the MF films. The phytochemicals in MF film could be attributed to the natural composition of mung beans. Venkatachalam and Nagarajan [42] observed that mung bean flour contains TPC and TFC and exhibits various antioxidant activities. Cortes-Rojas et al. [43] reported that clove essential oil is rich in polyphenols when compared to various spices. Gallic acid, flavonol glycosides, volatile phenolic oils (eugenol, acetyl eugenol), and tannins are the primary phenolic acids found in clove and clove oil. Lohani and Muthukumurappan [44] observed the effects of sonication on the TPC level in sorghum flour. They found that ultrasonication significantly increased the TPC level, and this was mainly because sonication released bound phenolics from the flour. In another study, Taha et al. [45] observed that sonication via strong cavitation and turbulence could help embed additives in the films. This is in accordance with the present study. Polyphenolics are the primary antioxidants in numerous plants and in plant-based films [46]. The present study observed various (ABTS, DPPH, and hydroxyl radical) radical scavenging activities and ferric reducing antioxidant power in the films (Table 3). The results show a significant level of radical scavenging activities. In general, MFCU and MFC films exhibited stronger activities against the DPPH radicals, followed by ABTS and hydroxyl radical scavenging. On the other hand, the MF film showed better performance against these radicals, and it was more effectively scavenging the ABTS radicals than hydroxyl or DPPH radicals. MF contains many essential amino acids, including leucine, lysine, phenylalanine, histidine, and valine, known as proton donors, that serve as excellent antioxidants [47,48]. Clove oil and sonication significantly affected free radical scavenging activities, and the phytochemical contents were directly correlated with the antioxidant properties of the films.

### 3.5. Antimicrobial Activities

The antimicrobial activities of mung bean flour films were tested using a quantitative method and examined their inhibitory effect of the film in the liquid culture (Table 4). In this study, all tested types of MF films showed potent activity against two strains of pathogenic bacteria, while the clove oil-infused films exhibited slightly better inhibition against the tested bacteria. Furthermore, the sonicated films (MFCU) had slightly improved inhibition against the microorganisms, although the differences to MFC were small. Several studies have reported that mung bean is a natural antimicrobial agent, which is mainly attributed to its phenolic compounds that create a low pH environment at the microbial cell membrane and induce cell membrane disruption through proton motive force [49]. Hafidh et al. [50] reported that mung bean’s antimicrobial activity was achieved by the presence of flavonoid compounds, particularly robinin, rutin, kaempferol, quercetin, isoquercetin, and kaempferol-7-O-rhanmnoside. Ahmad et al. [24] observed that the addition of essential oil (EO) could significantly improve the antimicrobial properties of the edible polymeric films. Clove EO is an excellent source of phenylpropenes, containing six carbon aromatic phenol groups and a three-carbon propene tail. Eugenol, isoeugenol, cinnamaldehyde, vanillin, and safrole are the predominant phenylpropenes present in clove EO. Phenylpropenes’ antimicrobial activity is determined by the aromatic ring’s type and number of substitutions. Nazzaro et al. [51] reported that the primary mode of action of EO’s antimicrobial activity is by altering proton motive force, increasing permeability, inducing coagulation, altering the membrane fatty acids, and limiting the ATP production in the microbial cells. This study found that MFCU and MFC exhibited stronger inhibition against Gram-negative bacteria (*C. jejuni*) than against Gram-positive bacteria (*S. aureus*). Hylgaard et al. [52] observed that eugenol and isoeugenol from the clove EO could inhibit gram-negative bacteria more than gram-positive bacteria. Furthermore, sonication also enhanced the antimicrobial activities by the controlled generation of reactive oxygen species in the microbial cells [53]. Ji et al. [54] found that the sonication of composite films more effectively inhibits the gram-negative bacteria than the gram-positive bacteria. This was mainly due to the strong interaction of *S. aureus* with the negatively charged reactive oxygen species (ROS) produced by sonication.

### 3.6. Lipid Oxidation Scavenging Abilities

**Figure 3:**
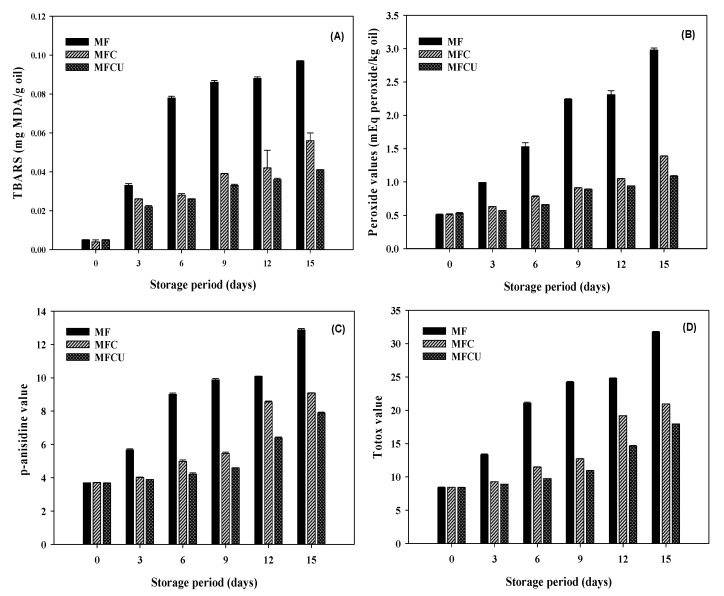
Lipid oxidation (TBARS (**A**), Peroxide value (**B**), p-anisidine value (**C**) and Totox value (**D**)) inhibition by mung bean flour-based films processed under different conditions. Note: TBARS represents Thiobarbituric acid reactive substances.

The control of lipid oxidation in olive oil by the edible films is shown in Figure 3. Olive oil was kept assessing lipid oxidation over 21 days, and samples of the edible films were added to the oil to assess their ability to control or suppress lipid oxidation. A clear increasing trend in indicators of lipid oxidation (TBARS, PV, p-anisidine, and totox values) in all samples without fluctuations indicates that lipid oxidation in the olive oil samples was adequate for testing the radical scavenging by immersed film samples. Overall, this study showed a controlling effect against lipid oxidation in olive oil. Malondialdehyde (MDA) is the primary product of lipid oxidation reactions, and the measurement of TBARS is the primary tool to determine them [55]. TBARS gradually increased during the extended storage of oil samples without immersed films. On the other hand, these values were suppressed, avoiding large increases by the added films. MF had the least control effect among the films, followed by MFC and MFCU. However, at the initial stage, the controlling effect by the films was not very detectable, and significant differences emerged as the storage period was extended. Phenolics are predominantly used to control the MDA in lipid-rich foods, and MF contains phenolics naturally; furthermore, the addition of clove oil and sonication treatment had released bound phenolics from the structure and thus effectively helped to control MDA [56]. Identifying the peroxide value (PV) for oil is equally essential as measuring MDA. A similar trend as for MDA was also observed for PV, namely a gradual increase of PV with time in all treatments. In particular, oil without an immersed film sample showed obvious peaks of PV, followed by oils with immersed film (Figure 3B). Among the films, MCFU controlled PV better than MCF and MF. Rapidly increasing PV was observed in the latter part of the storage test. The increase of MDA and PV levels in the oil was directly correlated with increased p-anisidine, which represents 2-alkenals and 2,4-dienals that are decomposition products of hydroperoxides [29]. A continuous increase in p-anisidine indicates that oxidation and decomposition of the oxidation product are progressing stably in the olive oil during the storage in all treatments (Figure 3C). The control samples had the highest p-anisidine levels, followed by MF, MFC, and MFCU. Overall, the level of p-anisidine was slightly controlled by the MF film and by clove oil-containing and sonication processed films. Besides, this study also checked the totox value, which is calculated from PV and p-anisidine levels for the oil. The totox represents the oxidation and stability of oil products based on both primary and secondary lipid oxidative products. MCFU film was the most effective in controlling the totox of the oil, followed by MFC and MF. The control sample showed totox exceeding 30%, indicating poor resistance to lipid oxidation. However, the added films in the oil significantly reduced the totox values during storage. Overall, the results show that MF film can control lipid oxidation. This might be attributed to natural antioxidants, while incorporating clove oil and sonication of MF film significantly improved the ability to prevent lipid oxidation. Pirsa and Asadi [57] reported that incorporation of antioxidant in the composite film could be able to retard the lipid oxidation by observing the free radicals and extending the shelf life of margarine.

## 4. Conclusions

This study found that the addition of clove oil and the use of a homogenization technique by sonication process improved the mung bean biodegradable film’s mechanical properties, induced interactions with phytochemicals, and improved antioxidant and antimicrobial activities. Films prepared with sonication incorporating clove oil had elevated levels of polyphenolics and flavonoids. These contributed to elevated ferric reducing antioxidant power and increased radical scavenging abilities, particularly for DPPH radical scavenging. In addition, this study also found that mung bean flour films inhibit Gram-negative pathogenic bacteria. Lipid oxidation was controlled most strongly when the films made of mung bean flour incorporated clove oil with sonication treatment. Overall, this study concluded that the use of mung bean flour as an active ingredient for biodegradable film has the broad potential for applications, especially in the food and pharmaceutical industries.

## Figures and Tables

**Table 1:** Physical properties of mung bean flour-based films processed under different conditions.

Treatments	Thickness (mm)	Bulk Density (g/cm^3^)	Moisture Content (%)	Solubility (%)	WVP10^−12^ (Kg Pa^−1^ s^−1^ m^−1^)
**MF**	0.191 ± 0.002 ^a^	0.857 ± 0.015 ^a^	12.477 ± 0.306 ^a^	31.0 ± 1.00 ^a^	6.03 ± 0.074 ^a^
**MFC**	0.196 ± 0.001 ^a^	0.943 ± 0.006 ^a^	10.28 ± 0.605 ^b^	29.66 ± 0.57 ^b^	5.60 ± 0.20 ^b^
**MFCU**	0.198 ± 0.001 ^a^	0.967 ± 0.015 ^a^	7.42 ± 0.366 ^c^	27.66 ± 0.57 ^c^	5.30 ± 0.10 ^b^

Note: MF represents mung bean flour films; MFC represents mung bean flour film incorporated with clove oil; MFCU represents the mung bean flour film incorporated with clove oil and processed with sonication. Data are shown as mean ± standard deviation. Different superscripts indicate significant differences.

**Table 2:** Mechanical properties of mung bean flour-based films processed under different conditions.

Treatments	Tensile Strength (MPa)	Elongation at Break (%)	Young’s Module (MPa)
**MF**	6.601 ± 0.193 ^b^	13.923 ± 0.731 ^c^	207.91 ± 0.64 ^c^
**MFC**	6.806 ± 0.047 ^b^	14.523 ± 0.160 ^b^	223.55 ± 4.15 ^b^
**MFCU**	7.285 ± 0.158 ^a^	15.077 ± 0.227 ^a^	246.64 ± 2.03 ^a^

Note: MF represents mung bean flour films; MFC represents mung bean flour film incorporated with clove oil; MFCU represents the mung bean flour film incorporated with clove oil and processed with sonication. Data are shown as mean ± standard deviation. Different superscripts indicate significant differences.

**Table 3:** Phytochemical and antioxidant activities of mung bean flour-based films processed under different conditions.

Treatments	Total Phenolic Content (µg GAE)/G	Total Flavonoid Content (µg CE)/G	Ferric Reducing Antioxidant Power (%)	ABTS + Radical Scavenging Activity (%)	DPPH Radical Scavenging Activity (%)	Hydroxyl Radical Scavenging Activity (%)
**MF**	17.90 ± 0.269 ^c^	13.63 ± 0.068 ^b^	68.33 ± 1.528 ^c^	65.00 ± 1.000 ^c^	55.66 ± 1.155 ^c^	62.00 ± 2.646 ^c^
**MFC**	47.57 ± 0.061 ^b^	29.17 ± 0.055 ^b^	80.33 ± 1.281 ^b^	70.00 ± 1.000 ^b^	75.33 ± 1.528 ^b^	69.00 ± 1.000 ^b^
**MFCU**	51.40 ± 0.115 ^a^	31.87 ± 0.166 ^a^	84.00 ± 1.000 ^a^	74.00 ± 2.000 ^a^	79.66 ± 1.155 ^a^	72.33 ± 2.082 ^a^

Note: MF represents mung bean flour films; MFC represents mung bean flour film incorporated with clove oil; MFCU represents mung bean flour film incorporated with clove oil and prepared with ultrasonication; GAE represent gallic acid equivalents; CE presents catechin equivalents; ABTS represents 2,2′-azino-bis (3-ethylbenzthiazoline-6-sulphonic acid; DPPH represents 2,2-diphenyl-1-picrylhydrazyl. Data are shown as mean ± standard deviation. Different superscripts indicate significant differences.

**Table 4:** Antimicrobial properties of mung bean flour-based films processed under different conditions.

Treatments	*S. aureus* Log (CFU/mL)	*C. jejuni* Log (CFU/mL)
**MF**	6.820 ± 0.177 ^a^	7.593 ± 0.163 ^a^
**MFC**	5.173 ± 0.152 ^b^	3.747 ± 0.122 ^b^
**MFCU**	4.440 ± 0.219 ^c^	3.090 ± 0.070 ^b^

Note: MF represents mung bean flour films; MFC represents mung bean flour film incorporated with clove oil; MFCU represents mung bean flour film incorporated with clove oil and prepared with sonication. Data are shown as mean ± standard deviation. Different superscripts indicate significant differences.

## Data Availability

Not applicable.

## References

[1] Montalvo-Paquini C., Avila-Sosa R., López-Malo A., Palou E. (2018). Preparation and characterization of proteinaceous films from seven Mexican common beans (*Phaseolus vulgaris* L.). J. Food Qual..

[2] Venkatachalam K., Keawpeng I., Thongbour P. (2017). Rheological and functional properties of wheat and green gram composite flours. Carpathian J. Food Sci. Tech..

[3] Moghadam M., Salami M., Mohammadian M., Khodadadi M., Emam-Djomeh Z. (2020). Development of antioxidant edible films based on mung bean protein enriched with pomegranate peel. Food Hydrocoll..

[4] Ganesan K., Xu B. (2018). A critical review on phytochemical profile and health promoting effects of mungbean (*Vigna radiata*). Food Sci. Hum. Wellness.

[5] Jom K.N., Frank T., Engel K.H. (2010). A metabolite profiling approach to follow the sprouting process of mung beans (*Vigna radiata*). Metabolomics.

[6] Bai Y., Xu Y., Chang J., Wang Z.Y., Yu Z. (2016). Bioactives from stems and leaves of mung beans. J. Funct. Foods.

[7] Chandrasiri S.D., Liyanage R., Vidanarachchi J.K., Weththasinghe P., Jayawardana B.C. (2016). Does processing have a considerable effect on the nutritional and functional properties of Mung bean (*Vigna radiata*). Proc. Food Sci..

[8] Cherng J.M., Chiang W., Chiang L.C. (2007). Immunomodulatory activities of edible beans and related constituents from soybean. Food Chem..

[9] Du M., Xie J., Gong B., Xu X., Tang W., Li X. (2018). Extraction, physicochemical characteristics and functional properties of Mung bean protein. Food Hydrocoll..

[10] Tharanathan R.N. (2003). Biodegradable films and composite coatings: Past, present and future. Trends Food Sci. Technol..

[11] Thompson R.C., Moore C.J., Vom Saal F.S., Swan S.H. (2009). Plastics, the environment and human health: Current consensus and future trends. Philos. Trans. R. Soc. B Biol. Sci..

[12] Nazmi N.N.M., Isa M.I.N., Sarbon N.M. (2020). Characterization of biodegradable protein-based films from gelatin alternative: A review. Int. Food Res. J..

[13] Khodaei D., Alvarez C., Mullen A.M. (2021). Biodegradable packaging materials from animal processing co-products and wastes: An overview. Polymers.

[14] Pirsa S., Sharifi A.K. (2020). A review of the application of bioproteins in the preparation of biodegradable films and polymers. J. Chem. Lett..

[15] Venkatachalam K., Lekjing S. (2020). A chitosan-based edible film with clove essential oil and nisin for improving the quality and shelf life of pork patties in cold storage. RSC Adv..

[16] Yao Y., Wang H., Wang R., Chai Y. (2019). Preparation and characterization of homogenous and enhanced casein protein-based composite films via incorporating cellulose microgel. Sci. Rep..

[17] Fattahi R., Seyedain-Ardabili M. (2021). A comparative study on the effect of homogenization conditions on the properties of the film-forming emulsions and the resultant films. Food Chem..

[18] Yildiz E., Ilhan E., Kahyaoglu L.N., Sumnu G., Oztop H.M. (2021). The effects of crosslinking agents on faba bean flour-chitosan-curcumin films and their characterization. Legume Sci..

[19] Mohamed S.A., El-Sakhawy M., El-Sakhawy M.A.M. (2020). Polysaccharides, protein and lipid-based natural edible films in food packaging: A review. Carbohydr. Polym..

[20] Choi J.E., Suk Oh M. (2009). Quality characteristics of mung bean starch gels with various hydrocolloids. J. Korean Soc. Food Cult..

[21] Jancikova S., Dordevic D., Tesikova K., Antonic B., Tremlova B. (2021). Active edible films fortified with natural extracts: Case study with fresh-cut apple pieces. Membranes.

[22] AOAC (2003). Official Methods of Analysis.

[23] Aydogdu A., Sumnu G., Sahin S. (2018). A novel electrospun hydroxypropyl methylcellulose/polyethylene oxide blend nanofibers: Morphology and physicochemical properties. Carbohydr. Polym..

[24] Ahmed J., Hiremath N., Jacob H. (2016). Antimicrobial, rheological, thermal properties of plasticized polylactide films incorporated with essential oils to inhibit staphylococcus aureus and campylobacter jejuni. J. Food Sci..

[25] Alberti A., Zielinski F.A.A., Zardo M.D., Demiate M.D., Nogueira M.I., Mafra I.L. (2014). Optimization of the extraction of phenolic compounds from apples using response surface methodology. Food Chem..

[26] Brand-Williams W., Cuvelier M.E., Berset C. (1995). Use of a free radical method to evaluate antioxidant activity. LWT Food Sci. Technol..

[27] Lee J.H., Lee J., Song K.B. (2015). Development of a chicken feet protein film containing essential oils. Food Hydrocoll..

[28] Halliwell N., Guteridge J.M., Aruoma O.I. (1987). The deoxyribose method: A simple “test-tube” assay for determination of rate constant for reactions of hydroxyl radicals. Anal. Biochem..

[29] Aydogdu A., Yildiz E., Aydogdu Y., Sumnu G., Sahin S., Ayhan Z. (2019). Enhancing oxidative stability of walnuts by using gallic acid loaded lentil flour based electrospun nanofibers as active packaging material. Food Hydrocoll..

[30] Ohl S., Klaseboer E., Khoo C.B. (2015). Bubbles with shock waves and ultrasound: A review. Interface Focus.

[31] Chandrapala J., Zisu B., Palmer M., Kentish S., Ashokkumar M. (2011). Effects of ultrasound on the thermal and structural characteristics of proteins in reconstituted whey protein concentrate. Ultrason. Sonochem..

[32] Aviles-Gaxiola S., Chuck-Hernandez C., Serna Saldivar O.S. (2018). Inactivation methods of trypsin inhibitor in legumes: A review. J. Food Sci..

[33] Jafarzadeh S., Alias A.K., Ariffin F., Mahmud S. (2018). Physico-mechanical and microstructural properties of semolina flour films as influenced by different sorbitol/glycerol concentrations. Int. J. Food Prop..

[34] Borah P.P., Das P., Badwaik L.S. (2017). Ultrasound treated potato peel and sweet lime pomace-based biopolymer film development. Ultrason. Sonochem..

[35] Cheng W., Chen J., Liu D., Ye X., Ke F. (2010). Impact of ultrasonic treatment on properties of starch film-forming dispersion and the resulting films. Carbohydr. Polym..

[36] Nascimento T.A., Calado V., Carvalho C.W.P. (2012). Development and characterization of flexible film based on starch and passion fruit mesocarp flour with nanoparticles. Food Res. Int..

[37] Stoleru E., Vasile C., Irimia A., Brebu M. (2021). Towards a bioactive food packaging: Poly(lactic acid) surface functionalized by chitosan coating embedding clove and argan oils. Molecules.

[38] Kocakulak S., Sumnu G., Sahin S. (2019). Chickpea flour-based biofilms containing gallic acid to be used as active edible films. J. Appl. Polym. Sci..

[39] Chieng B., Ibrahim N., Yunus W., Hussein M. (2013). Poly(lactic acid)/poly(ethylene glycol) polymer nanocomposites: Effects of graphene nanoplatelets. Polymers.

[40] Warren F.J., Gidley M.J., Flanagan B.M. (2016). Infrared spectroscopy as a tool to characterize starch ordered structure-a joint FTIR–ATR, NMR, XRD and DSC study. Carbohydr. Polym..

[41] Hoque M.S., Benjakul S., Prodpran T. (2020). Effect of heat treatment of film-forming solution on the properties of film from cuttlefish (Sepia pharaonic) skin gelatin. J. Food Eng..

[42] Venkatachalam K., Nagarajan M. (2017). Physicochemical and sensory properties of savory crackers incorporating green Gram to partially of wholly replace wheat flour. Ital. J. Food Sci..

[43] Cortes-Rojas D.F., Fernandes de Souza R.C., Oliveira P.W. (2014). Clove (*Syzygium aromaticum*): A precious spice. Asian Pac. J. Trop. Biomed..

[44] Lohani C.U., Muthukumarappan K. (2021). Study of continuous flow ultrasonication to improve total phenolic content and antioxidant activity in sorghum flour and its comparison with batch ultrasonication. Ultrason. Sonochem..

[45] Taha A., Ahmed E., Ismaiel A., Ashokkumar M., Xu X., Pan S., Hu H. (2020). Ultrasonic emulsification: An overview on the preparation of different emulsifiers-stabilized emulsions. Trends Food Sci. Technol..

[46] Shi Z., Yao Y., Zhu Y., Ren G. (2016). Nutritional composition and antioxidant activity of twenty mung bean cultivars in China. Crop J..

[47] Gul O., Saricaoglu F.T., Besir A., Atalar I., Yazici F. (2018). Effect of ultrasound treatment on the properties of nano-emulsion films obtained from hazelnut meal protein and clove essential oil. Ultrason. Sonochem..

[48] Yi-Shen Z., Shuai S., FitzGerald R. (2018). Mung bean proteins and peptides: Nutritional, functional and bioactive properties. Food Nutr. Res..

[49] Randhir R., Lin Y., Shetty K. (2004). Stimulation of phenolics, antioxidant and antimicrobial activities in dark germinated mung bean sprouts in response to peptide and phytochemical elicitors. Process Biochem..

[50] Hafidh R.R., Abdulamir A.S., Vern L.S., Bakar A.F., Abas F., Jahanshiri F., Sekawi Z. (2011). Novel in-vitro antimicrobial activity of *Vigna radiata* (L.) R. Wilczek against highly resistant bacterial and fungal pathogens. J. Med. Plants Res..

[51] Nazzaro F., Fratianni F., Martino D.L., Coppola R., Feo V. (2013). Effect of essential oils on pathogenic bacteria. Pharmaceuticals.

[52] Hyldgaard M., Mygind T., Rikke T. (2012). Essential oils in food preservation: Mode of action, synergies, and interactions with food matrix components. Front. Microbiol..

[53] Wang Y., Xu Y., Dong S., Wang P., Chen W., Lu Z., Ye D., Pan B., Wu D., Vecitis D.C. (2021). Ultrasonic activation of inert poly (tetrafluoroethylene) enables piezocatalytic generation of reactive oxygen species. Nat. Commun..

[54] Ji T., Zhang R., Dong X., Sameen E.D., Ahmed S., Li S., Liu Y. (2020). Effect of ultrasonication time on the properties of polyvinyl alcohol/sodium carboxymethyl cellulose/nano-Zno/multilayer graphene nanoplatelet composite films. Nanomaterials.

[55] Zeb A., Ullah F. (2016). A simple spectrophotometric method for the determination of thiobarbituric acid reactive substances in fried fast foods. J. Anal. Methods Chem..

[56] Shahidi F., Ambigaipalan P. (2015). Phenolics and polyphenolics in foods, beverages and spices: Antioxidant activity and health effects—A review. J. Func. Foods.

[57] Pirsa S., Asadi S. (2021). Innovative smart and biodegradable packaging for margarine based on a nano composite polylactic acid/lycopene film. Food Addit. Contam. Part A.

